# Editorial: Head and neck cancer in the elderly - vol II

**DOI:** 10.3389/fonc.2025.1591925

**Published:** 2025-03-28

**Authors:** Petr Szturz, Jan B. Vermorken

**Affiliations:** ^1^ Department of Oncology, Lausanne University Hospital (CHUV), University of Lausanne (UNIL), Lausanne, Switzerland; ^2^ Department of Medical Oncology, Antwerp University Hospital, Edegem, Belgium; ^3^ Faculty of Medicine and Health Sciences, University of Antwerp, Antwerp, Belgium

**Keywords:** geriatric medicine, epidemiology, chemoradiotherapy, systemic treatment, radiotherapy, immunotherapy, thyroid cancer

Older age represents one of the most common challenging patient factors encountered in clinical practice. Advanced chronological age is a well-known prognostic factor but has limited predictive value. This was highlighted in the landmark Pignon meta-analysis, which showed that patients over 70 years, diagnosed with squamous cell carcinoma of the head and neck (SCCHN), did not seem to derive a survival advantage from the addition of concomitant chemotherapy to radiotherapy (1). This finding may be erroneously interpreted as evidence that patients over 70 years of age should not receive chemotherapy in this setting, as if age were a negative predictor of chemotherapy efficacy. However, this is incorrect. Chemotherapy retains its anticancer potential in older patients, who can still benefit from its administration, albeit with a generally worse overall prognosis. The explanation lies in the progressively increasing risk of competing mortality due to other causes.

In fact, this observation was also reported in the Pignon meta-analysis, but it may have been overlooked despite its fundamental importance. There are two principal contributors to competing mortality: first, the increased comorbidity burden in elderly patients, and second, chemotherapy-related toxicity. While it may be difficult to distinguish between these two causes, this distinction provides valuable insight into the optimal management of older patients. It underscores the importance of baseline clinical assessment to identify fit elderly patients, in whom chemotherapy is likely to provide a survival benefit, versus frail patients, in whom the risk-benefit ratio is unfavourable, with vulnerable patients falling somewhere in between. This aligns with findings from population-based cross-sectional registry studies, which confirm a survival benefit for chemotherapy in elderly patients. Notably, most of these positive studies accounted for comorbidity burden, as determined by the Charlson-Deyo comorbidity score (2).

Despite these insights, concerns about increased toxicity in elderly patients are legitimate, even in those deemed fit. Due to the physiological decline in organ function, older adults are at an elevated risk of developing various adverse events (3–5). However, this should not deter clinicians from administering chemotherapy but rather encourage closer monitoring. This reasoning is particularly relevant to the age span between 65 or 70 (depending on the predefined lower limit of elderly) and 80 years, impacting thus the majority of older patients during the period when most of them remain alive. However, with increasing life expectancy, the number of octogenarians, nonagenarians, and even centenarians is rising. For these patients, clinical trial data are scarce because they are rarely enrolled in studies, and dedicated trials are practically non-existent. Given the further decline in organ function in the latter age groups, these patients are presumed to be at an even higher risk of complications. This assumption must be weighed against the potential benefits of each anticancer treatment.

For example, adding concurrent chemotherapy to radiotherapy improves 5-year overall survival by approximately 10%, with a noticeable separation of the survival curves emerging around 2 years after treatment initiation (1, 6, 7). However, many of the oldest patients may not live long enough to benefit from this improvement, considering that the average life expectancy in the European Union, roughly corresponding to the median age at death, was estimated at 81.5 years in 2023, with geographic variations (8). While our goal is to offer optimal therapy to these patients, significant knowledge gaps remain, not only regarding the oldest individuals but the elderly population in general. The need to better understand and characterize this patient group was also the primary motivation behind the two article collections published in this Research Topic. The first was completed two years ago, and we are now pleased to present a second edition featuring four new articles: two focusing on chemoradiotherapy, one on immunotherapy, and one on thyroid cancer, a topic not covered in the previous edition.

Yasuda et al. conducted a survey within the Head and Neck Cancer Study Group of the Japan Clinical Oncology Group (JCOG) to assess practice patterns in administering cisplatin concurrently with radiotherapy in elderly patients. The investigators found that the primary factors influencing decisions about this therapy were renal function, measured by glomerular filtration rate (GFR), and performance status (PS), rather than chronological age itself. Specifically, most respondents agreed that high-dose, three-weekly cisplatin could be given to patients aged 65–74 years with PS 0–1 and an estimated GFR (eGFR) ≥60 ml/min/1.73m². However, they did not recommend this regimen for patients aged ≥75 years with PS 2, ≥80 years with PS 1, or ≥65 years with an eGFR <60 ml/min/1.73m². Regarding weekly low-dose cisplatin administration, the consensus was that it should not be given to patients aged ≥75 years with PS 2, ≥70 years with an eGFR <50 ml/min/1.73m², or ≥65 years with an eGFR <40 ml/min/1.73m². These findings further reinforce the common understanding of the need for caution in the oldest patients.

The second paper, by Winkler et al., examined real-world data on newly diagnosed non-metastatic SCCHN treated with curative intent radiotherapy or chemoradiotherapy, both in the definitive and adjuvant settings. Between 2010 and 2021, 71 patients aged 76 years or older were included and subsequently followed for a median of 18 months. Importantly, data on comorbidities, comprising the Charlson-Deyo comorbidity score, and functional status, assessed using the Barthel index, were recorded. One-quarter of the cohort had human papillomavirus-positive oropharyngeal cancer. The 3-year overall survival and progression-free survival rates were 72% and 46%, respectively. Nearly half of the cohort (48%) received standard therapy according to current guidelines, while deviations were primarily due to medical reasons to prevent the toxicity of cisplatin-based chemotherapy. Two-thirds of patients completed therapy as intended, and there were no treatment-related deaths.

In the third contribution, Salvestrini et al. evaluated the outcomes of immunotherapy in the elderly population. The authors conducted a systematic review and meta-analysis including subgroups of patients over 65 years from randomised clinical trials in the locoregionally advanced and recurrent and/or metastatic settings. The primary objectives were overall and progression-free survival, while secondary endpoints involved safety and quality of life. The beneficial impact of immunotherapy on both survival measures was comparable between older and younger participants. Age-specific toxicity data were available only from the CheckMate-141 study, which reported an acceptable 13% rate of severe acute adverse events, similar to the overall study population, suggesting that immunotherapy remains a viable option for older patients. Of note, among 40 reports assessed for eligibility, the majority were excluded due to missing data on response, survival, or toxicity in the elderly subgroup. Only four and seven trials were eligible for the efficacy and safety analyses, respectively, highlighting the insufficient reporting on the elderly population and calling for urgent improvements in this area.

Finally, Dou et al. analysed data from the Global Burden of Disease database to estimate epidemiological trends in thyroid cancer from the 1990s to the 2010s. Paralleled by an overall rise in incidence, the prevalence grew from approximately 6 million cases in 1990 to 18 million cases in 2019, primarily due to earlier detection resulting from improved diagnostics and greater health awareness, but also to increased exposure to environmental and lifestyle risk factors. The highest incidence, which remained consistent over the examined three decades, was noted in China, the US, and India. Mortality was highest among individuals aged 65–79 years in both genders, likely influenced by competing risks of death. The authors also evaluated DALYs (disability-adjusted life-years), YLL (years of life lost), and YLD (years lived with disability), finding that these parameters increased over time, despite declining age-standardised rates. In 2019, DALYs peaked in women aged 60–64 years and 65–69 years and in men aged 55–59 years.

In conclusion, we hope that this Research Topic will increase interest in geriatric oncology and stimulate further research initiatives in various unresolved areas. Above all, selecting the most appropriate therapy for elderly patients is paramount. Fit elderly people should be treated like their younger counterparts. However, a better selection process is needed as well as increased vigilance to mitigate the risk of toxicity in such patients. It cannot be said enough that close collaboration with dedicated geriatricians is essential.
